# Quality of life correlation with socioeconomic status in Korean hepatitis-B patients: a cross sectional study

**DOI:** 10.1186/s12955-015-0251-3

**Published:** 2015-05-12

**Authors:** Seung Ju Kim, Kyu-Tae Han, Seo Yoon Lee, Eun-Cheol Park

**Affiliations:** Department of Public Health, Graduate School, Yonsei University, Seoul, Republic of Korea; Institute of Health Services Research, Yonsei University, Seoul, Republic of Korea; Department of Health Policy and Management, Graduate School of Public Health, Yonsei University, Seoul, Republic of Korea; Department of Preventive Medicine, Yonsei University College of Medicine, 50 Yonsei-ro, Seodaemun-gu, Seoul, 120-752 Republic of Korea

## Abstract

**Background:**

In Korea, more than two-thirds of hepatocellular carcinoma patients are hepatitis B virus (HBV) surface antigen-seropositive. The effects of HBV infection on health-related quality of life (HRQoL) are important aspects in the overall management of HBV infection. However, other effects of other parameters on HBV patient HRQoL remain unknown and require clarification. Our study evaluated HRQoL in hepatitis-B patients, according to socioeconomic status.

**Methods:**

We used community health survey data to analyze the relationship between HRQoL of HBV^+^ patients according to socioeconomic status. We used propensity score matching (Ratio = 1:5) to balancing the patients and general population. Final analytic sample consisted of 7,098 hepatitis B patients and compared group (35,490 general populations). We examined the HRQoL in HBV^+^ patients (n = 7,098), stratified by socioeconomic status, compared with general populations, using the EuroQOL visual analog scale (EQ-VAS) and EQ-5D questionnaires. We used the Chi-square test and ANOVA to compare demographic variables. Multiple linear regression analysis identified associations between demographic variables and HRQoL.

**Results:**

Participants with hepatitis B numbered 7,098 (16.7 %) of the study population. HRQoL was lower in hepatitis-B patients compared to the general population (EQ-VAS: −0.985, p = 0.0004; EQ-5D: −0.673, p = 0.0003). According to occupation type, clerks (EQ-VAS: −2.628, p = 0.0030; EQ-5D: −0.802, p = 0.0099) and managers and professionals (EQ-VAS: −1.518, p = 0.0356) had the lowest HRQoLs. Higher family income and education level groups had lower HRQoLs compared to the general population.

**Conclusions:**

Patients from higher socioeconomic status groups had HRQoLs that were more affected by hepatitis B. Thus, we require more accurate information about the disease to develop appropriate patient management guidelines. This will facilitate formulating policies and management strategies that alleviate HRQoL declines in HBV^+^ patients.

## Background

The global burden of chronic diseases contributes to a major public health challenge that undermines social and economic development worldwide. An estimated 39 million deaths occurred globally in 2008 that were due to noncommunicable diseases such as cardiovascular disease (48 %), cancer (21 %), chronic respiratory disease (12 %), and diabetes (3.5 %) [1]. Globally, of these diseases, cancer is increasingly responsible for a large proportion of deaths, and two of the most common sites for mortality are the liver and stomach [2].

In Korea, cancer is the leading cause of mortality, and the liver is second most common primary tumor site that causes death. Liver cancer prevalence in Korea also increased during a recent 10-year span, from 29.0 % in 2001 to 32.9 % in 2011 [3]. Hepatitis B virus (HBV) infection is a leading risk factor for developing hepatocellular carcinoma globally, and in Korea more than two-thirds (74.2 %) of liver cancer cases tested positive for circulating HBV surface antigen [4]. The most important approach to preventing HBV infection is childhood vaccination, and the World Health Organization recommended that all countries introduce a policy of universal HBV vaccination in 1991; as of 2011, this policy had been introduced nationwide in 180 countries, with coverage approaching 80 % [5]. Regardless of the mode of infection, HBV infection remains highly endemic in the Western Pacific region [6, 7]. In the Korean general population, the HBV surface antigen-positive prevalence rate has decreased from 10 % in the 1980s to 3.8 % in 2007, following the introduction of HBV vaccination [8]. However, a previous study suggested that this decreased HBV prevalence is limited to the young population and that HBV infection persists in the middle-aged and older populations [9]. And HBV seems to have more important role than hepatitis C in Korea, with respect to promoting the pathogenesis of specific hematologic malignancies, and is associated with increased risk of lymphomas and acute myeloid leukemia [10]. Thus, preventing and managing HBV infection are important considerations in Korean public health.

Measuring health-related quality of life (HRQoL) is commonly used to gauge outcomes in many other diseases. HRQoL is often linked to health, and components of happiness and satisfaction with life are emphasized. The impact of liver disease on HRQoL is multifaceted. In general, the presence of liver disease seems to negatively affect HRQoL. Previous studies identified a close association between hepatitis C, depression, and HRQoL [11–16]. Also, the association between HBV infection and quality of life was documented [17–21]. Despite increasing the interest in assessing HRQoL in different types of liver disease, research into other factors that can affect HRQoL in HBV^+^ patients is lacking. Not only is there a need for studies on general aspects of HBV and HRQoL, but there is also need for assessing the effects of socioeconomic status on patient HRQoL. Especially, Korea has traditional values and unique working environments that may affect patient HRQoL [22]. Our study evaluated the HRQoL associated with hepatitis B patients using a visual analogue scale and the EQ-5D survey. We further explored HRQoL differences in HBV patients according to socioeconomic status.

## Methods

### Study population

The data used in this study were obtained from the 2011–2012 Community Health Survey administered by the Korea Centers for Disease Control and Prevention, which was designed to facilitate interprovincial comparisons. The Community Health Survey was administered by investigators who conducted one-on-one visits and interviews targeting adults ≥19-years-old in 253 health centers nationwide, starting in 2008. We excluded hepatitis C patients and participants with incomplete HRQoL information; HBV+ patients were defined as people who were ever diagnosed with hepatitis B, and those histories of HBV+ in people were investigated by interview.

In the data used in our study, only 2.5 % of participants had hepatitis B, in order to achieve fair comparison, it was necessary to balancing the patients and general population. Thus, we refer to the two groups as the hepatitis B patients and comparison groups and assume that a patient is matched to one or more comparison general population. Propensity score matching (Ratio = 1:5) was used in our paper by matching participants on a number of covariates that may affect in each participant group. This approach is that participants have an underlying propensity to be in one group or another. By matching based on preexisting characteristics, provides a way of statistically controlling the variation in these characteristics to minimize selection effects. A total analytic sample consisted of 7,098 hepatitis B patients and compared group (35,490 general populations) came from similar characteristics after matching the propensity score.

### Variables

The outcome variables were evaluated using the EuroQOL visual analog scale (EQ-VAS) and EQ-5D. The EQ-VAS is a self-rated health questionnaire presented as a vertical visual analog scale, where the endpoints are labeled ‘best imaginable health state’ and ‘worst imaginable health state’. Participants completed the questionnaires on the study day. Scores ranged from 0 to 100 and the responses were used as a quantitative measure of participant self-rated health. The EQ-5D is a questionnaire that characterized health in five domains, i.e., mobility, self-care, usual activities, pain/discomfort, and anxiety/depression on three levels to define HRQoL values.

Other independent variables considered in the analysis were occupation type, marital status, sex, smoking status, alcohol consumption, stress, number of chronic disease, education, income, age, and year. Occupations were divided into managers and professionals, clerks, service and sales workers, skilled agricultural, forestry and fishery workers, trade workers and elementary occupation, and not working. Marital status was classified as “Single”, “Separated/Divorced/Bereavement” or “Married”, and smoking status as “Smoker”, “Ex-smoker”, or “Non-smoker”. Alcohol consumption was defined as “Yes” or “No” following the question: “Have you ever drank alcohol in the past one year?” Stress was classified as those who reported feeling stress or those who did not. Chronic disease classified to three groups according to a number of disease which was included asthma, diabetes, hypertension, dyslipidemia and thyroid lesion. People who do not have these chronic diseases were classified as ‘0’. All disease was defined as people who ever had been diagnosed each disease. Education level was classified as elementary school, middle school, high school, or college graduate. Family income was classified into four groups based on quartiles (Q1: lowest income/Q4: highest income), and age was classified into 19–29 years, 30–39 years, 40–49 years, 50–59 years, and ≥60-years-old.

### Statistical analyses

All analyses were conducted using SAS version 9.3 (SAS Institute Inc., Cary, NC). We assigned weight to sampling results to convey an accurate representation of the whole nation. Baseline demographic and clinical characteristics were compared using the Chi-squared test. We next compared the average EQ-VAS and EQ-5D scores according to the independent variables, using ANOVA. In the fully adjusted model, all variables were entered simultaneously. Multiple linear regression analysis was performed to identify associations of these variables with HRQoL (normal population vs hepatitis B), while controlling for potential confounding variables. Additionally, we performed subgroup analyses based on occupation, education, and family income. P-values <0.05 were considered indicative of statistically significant differences.

## Results

In our study, a total of 7098 (16.7 %) of participants were HBV-positive. In occupation type, not working (33.4 %) and trade workers and elementary occupations (24.6 %) comprised the highest HBV^+^ frequencies. A total of 830 (11.7 %) HBV^+^ participants were single, 982 (13.8 %) participants were separated, divorced or bereaved, and 5,286 (74.4 %) were married. And male participants that were HBV^+^ (n = 3,947; 55.6 %) were frequent than females (n = 3,151; 44.4 %). Regarding smoking status, almost half of HBV^+^ participants were non-smokers 3,771 (53.1 %), smokers numbered 1,798 (25.3 %), and ex-smokers were 1,529 (21.5 %). More participants with HBV were alcohol consumers (n = 4,844; 68.2 %) than non-consumers (n = 2,254; 31.8 %). Of HBV^+^ participants, 2,216 (31.2 %) reported feeling stressed. Almost two-thirds of participants (n = 4,357; 61.4 %) didn’t report concurrent chronic diseases (Table 1).

**Table 1:** General characteristics of participants (N, %)

	Hepatitis B patients		General population		Total	p-value
**Occupation**						
Managers and professionals	781	(11.0)	3901	(11.0)	4,682	0.6203
Clerks	590	(8.3)	2,955	(8.3)	3,545	
Service and sales workers	1,024	(14.4)	5,254	(14.8)	6,278	
Skilled agricultural, forestry and fishery workers	591	(8.3)	3,060	(8.6)	3,651	
Trade workers and elementary occupations	1,744	(24.6)	8,385	(23.6)	10,129	
Not working	2,368	(33.4)	11,935	(33.6)	14,303	
**Marital status**						
Single	830	(11.7)	3,956	(11.1)	4,786	0.3920
Seperated/Divorced/Bereavement	982	(13.8)	4,890	(13.8)	5,872	
Married	5,286	(74.5)	26,644	(75.1)	31,930	
**Sex**						
Male	3,947	(55.6)	19,778	(55.7)	23,725	0.8512
Female	3,151	(44.4)	15,712	(44.3)	18,863	
**Smoking status**						
Smoker	1,798	(25.3)	8,846	(24.9)	10,644	0.7434
Ex-smoker	1,529	(21.5)	7,736	(21.8)	9,265	
Non-smoker	3,771	(53.1)	18,908	(53.3)	22,679	
**Alcohol consumption**						
Yes	4,844	(68.2)	24,407	(68.8)	29,251	0.3822
No	2,254	(31.8)	11,083	(31.2)	13,337	
**Stress**						
Yes	2,216	(31.2)	11,075	(31.2)	13,291	0.9813
No	4,882	(68.8)	24,415	(68.8)	29,297	
**Chronic disease**						
0	4,357	(61.4)	22,590	(63.7)	26,947	
1	1,791	(25.2)	8,590	(24.2)	10,381	
≥2	950	(13.4)	4,310	(12.1)	5,260	
**Education**						
Elementary school	1,425	(20.1)	7,043	(19.8)	8,468	0.6587
Middle school	1,049	(14.8)	5,411	(15.2)	6,460	
High school graduate	2,666	(37.6)	13,416	(37.8)	16,082	
College graduate	1,958	(27.6)	9,620	(27.1)	11,578	
**Family income**						
Q1	1,697	(23.9)	8,418	(23.7)	10,115	0.9870
Q2	2,152	(30.3)	10,813	(30.5)	12,965	
Q3	1,953	(27.5)	9,765	(27.5)	11,718	
Q4	1,296	(18.3)	6,494	(18.3)	7,790	
**Age**						
20-29	542	(7.6)	2,645	(7.5)	3,187	0.9257
30-39	1,127	(15.9)	5,575	(15.7)	6,702	
40-49	1,647	(23.2)	8,177	(23.0)	9,824	
50-59	1,834	(25.8)	9,191	(25.9)	11,025	
60≤	1,948	(27.4)	9,902	(27.9)	11,850	
**Year**						
2011	3,578	(50.4)	17,917	(50.5)	21,495	0.9068
2012	3,520	(49.6)	17,573	(49.5)	21,093	
**Total**	7,098	(16.7)	35,490	(83.3)	42,588	<.0001

The average EQ-VAS and EQ-5D scores were higher in general population than in the HBV^+^ group (HBV^+^: EQ-VAS: 70.2; EQ-5D: 93.1/general population: EQ-VAS: 71.2; EQ-5D: 93.8). Regarding occupation type, the managers and professionals category scored highest on the EQ-VAS and EQ-5D, in both the HBV^+^ group and in the general population. Regarding marital status, ‘single’ scored highest in both the HBV^+^ group and in the general population. As education increased, EQ-VAS and EQ-5D scores gradually increased. A similar trend was found in the family income analyses, in which high income family had higher HRQoL scores in both the HBV^+^ group and in the general population, but scores were higher in the general population (Table 2).

**Table 2:** Relationships of quality of life with demongraphic characteristics

	Hepatitis B patients						General population					
	EQ-VAS			EQ-5D			EQ-VAS			EQ-5D		
	Means	SD	P-Value	Means	SD	P-Value	Means	SD	P-Value	Means	SD	P-Value
**Occupation**												
Managers and professionals	74.9	15.3	<.0001	97.2	6.1	<.0001	76.2	14.8	<.0001	97.9	5.4	<.0001
Clerks	73.1	17.3		97.1	6.6		75.3	14.8		98.2	5.1	
Service and sales workers	72.5	16.5		95.7	7.3		73.2	16.6		96.3	7.6	
Skilled agricultural, forestry and fishery workers	67.7	18.0		91.1	12.8		68.8	18.3		91.9	11.2	
Trade workers and elementary occupations	71.6	17.3		95.3	8.6		73.0	16.8		96.3	7.8	
Not working	66.6	20.9		88.5	14.9		67.1	20.1		88.9	15.4	
**Marital status**												
Single	71.3	17.8	0.0107	95.9	9.0	<.0001	73.7	16.9	<.0001	96.8	8.7	<.0001
Seperated/Divorced/Bereavement	64.5	20.5		87.3	15.0		64.8	20.0		87.9	15.1	
Married	71.1	18.1		93.7	10.9		72.1	17.5		94.4	10.8	
**Sex**												
Male	71.6	18.0	0.0107	94.6	10.7	0.0002	72.7	17.4	<.0001	95.0	10.9	<.0001
Female	68.5	19.1		91.2	12.3		69.4	18.6		92.2	12.2	
**Smoking status**												
Smoker	70.0	18.4	0.0054	94.3	11.0	0.1629	70.9	17.8	<.0001	95.2	10.2	0.0005
Ex-smoker	71.5	18.5		93.5	11.3		72.4	18.0		93.5	12.6	
Non-smoker	69.9	18.7		92.3	11.9		70.9	18.1		93.2	11.6	
**Alcohol consumption**												
Yes	71.5	17.5	0.0079	94.5	10.0	0.0027	72.7	17.0	<.0001	95.5	9.3	<.0001
No	67.5	20.4		90.1	14.0		68.1	19.7		90.0	14.8	
**Stress**												
Yes	63.8	20.1	<.0001	89.8	14.2	<.0001	65.3	19.5	<.0001	90.8	14.1	<.0001
No	73.2	17.0		94.6	9.8		73.9	16.6		95.1	9.9	
**Chronic disease**												
0	72.0	17.5	<.0001	95.0	9.6	<.0001	73.2	17.1	<.0001	95.6	9.4	<.0001
1	68.9	19.5		91.3	12.7		68.9	18.8		91.5	13.4	
≥2	64.6	20.2		87.6	15.0		65.7	19.5		88.5	14.8	
**Education**												
Elementary school	63.3	20.0	<.0001	85.8	15.6	<.0001	64.0	20.2	<.0001	86.2	15.5	<.0001
Middle school	68.4	19.5		91.5	12.4		69.4	18.7		92.0	12.7	
High school graduate	72.1	17.8		94.9	9.4		72.9	17.1		95.8	9.3	
College graduate	73.9	16.3		96.7	7.2		75.4	15.3		97.5	6.4	
**Family income**												
Q1	63.2	20.5	<.0001	86.4	15.7	<.0001	64.1	20.2	<.0001	87.2	15.5	<.0001
Q2	70.2	17.9		93.7	10.3		71.5	17.6		94.3	10.6	
Q3	73.3	16.8		96.1	8.0		74.0	16.3		96.5	7.9	
Q4	74.9	16.6		96.3	7.6		76.0	15.3		97.1	7.4	
**Age**												
20-29	73.3	17.7	0.0202	97.5	6.0	<.0001	75.3	16.4	<.0001	98.0	5.6	<.0001
30-39	71.3	17.3		96.4	7.8		73.5	16.0		97.7	5.7	
40-49	72.9	17.2		95.6	8.7		73.5	16.7		96.5	8.2	
50-59	70.6	18.8		93.3	11.2		71.9	17.9		94.3	10.9	
60≤	66.2	19.6		87.6	14.7		66.4	19.7		87.7	15.1	
**Year**												
2011	69.0	18.9	<.0001	92.8	11.9	0.0063	70.1	18.5	<.0001	93.4	11.9	<.0001
2012	71.5	18.1		93.4	11.3		72.4	17.4		94.1	11.2	
**Total**	70.2	18.6		93.1	11.6		71.2	18.0		93.8	11.5	

In regression model analysis, HBV^+^ patients had lower EQ-VAS (−0.985, p = 0.0004) and EQ-5D (−0.673, p < 0.0001) scores than the general population. Quality of life scores were lower in low academic status (EQ-VAS: −4.869, p < 0.0001; EQ-5D: −4.928, p < 0.0001). A similar trend was found in family income, wherein lower family income has the lowest HRQoL scores (EQ-VAS: −5.889, p < 0.0001; EQ-5D: −2.355, p < 0.0001). According to age, younger people scored highest on the EQ-5D (4.004, p < 0.0001) referring to people aged ≥60-years-old. According to occupation, managers and professionals scored highest on quality of life compared to not-working participants (EQ-VAS: 3.418, p < 0.0001; EQ-5D: 3.518, p < 0.0001). Regarding marital status, separated, divorced, and bereaved participants had lower scores on the EQ-VAS (−2.395, p < 0.0001) and EQ-5D (−2.018, p < 0.0001) compared to married participants (Table 3).

**Table 3:** Regression model analysis results of EQ-VAS and EQ-5D

		EQ-VAS		EQ-5D	
		Estimate	p-value	Estimate	p-value
**Hepatitis B**					
Yes		−0.985	0.0004	−0.673	<.0001
No		Ref	-	Ref	-
**Occupation**					
Managers and professionals		3.418	<.0001	3.518	<.0001
Clerks		2.911	<.0001	3.421	<.0001
Service and sales workers		2.809	<.0001	3.552	<.0001
Skilled agricultural, forestry and fishery workers		2.048	<.0001	3.793	<.0001
Trade workers and elementary occupations		2.746	<.0001	3.950	<.0001
Not working		Ref	-	Ref	-
**Marital status**					
Single		−0.486	0.219	−0.497	0.0073
Seperated/Divorced/Bereavement		−2.395	<.0001	−2.018	<.0001
Married		Ref	-	Ref	-
**Sex**					
Male		2.245	<.0001	0.690	<.0001
Female		Ref	-	Ref	-
**Smoking status**					
Smoker		−2.870	<.0001	−0.372	0.0179
Ex-smoker		−0.606	0.0711	−0.271	0.1316
Non-smoker		Ref	-	Ref	-
**Alcohol consumption**					
Yes		0.937	0.0002	1.399	<.0001
No		Ref	-	Ref	-
**Stress**					
Yes		−8.368	<.0001	−4.105	<.0001
No		Ref	-	Ref	-
**Chronic disease**					
0		Ref	-	Ref	-
1		−2.150	<.0001	−1.213	<.0001
≥2		−4.617	<.0001	−2.848	<.0001
**Education**					
Elementary school		−4.869	<.0001	−4.928	<.0001
Middle school		−3.003	<.0001	−2.500	<.0001
High school graduate		−0.786	0.0037	−0.588	<.0001
College graduate		Ref	-	Ref	-
**Family income**					
Q1		−5.889	<.0001	−3.587	<.0001
Q2		−2.355	<.0001	−0.825	<.0001
Q3		−1.278	<.0001	−0.340	0.0077
Q4		Ref	-	Ref	-
**Age**					
20-29		0.914	0.1106	4.004	<.0001
30-39		−1.253	0.0032	3.044	<.0001
40-49		−0.440	0.2496	2.269	<.0001
50-59		0.280	0.4208	1.983	<.0001
60≤		Ref	-	Ref	-
**Year**					
2011		−2.056	<.0001	−0.431	0.0001
2012		Ref	-	Ref	-

Also, to compare HBV^+^ participants with general population in each variable group, we performed sub-group analyses by considering each variable such as occupation type, education, and family income. In the results of sub-group analysis by occupation types, clerks scored the lowest on the EQ-VAS (−2.628, p = 0.0030), followed by managers and professionals (−1.518, p = 0.0356). Similar results were observed with the EQ-5D, on which trade workers and elementary occupations (−0.828, p = 0.0007), following by clerks (−0.802, p = 0.0099) and not-working (−0.743, p = 0.0251). Regarding educational level, HBV^+^ patients that were college graduates had the lowest score on the EQ-VAS (−1.606, p = 0.0003) high school graduates scored lowest on the EQ-5D (−0.968, p < 0.0001). There was a trend of decreased EQ-VAS scores in participants with higher academic status. According to family income, quartile-1 (Q1) HBV^+^ patients had lowest EQ-VAS scores (−1.302, p = 0.0683) but, it is not statistically significant. Q2 and Q4 patients scored the lowest on the EQ-5D (Q2: −0.681, p = 0.0155; Q4: −0.872, p = 0.0003) (Table 4).

**Table 4:** Sub-group analysis of EQ-VAS and EQ-5DS scale according to occupation, education and family income level in hepatitis B (unit: coefficient, p = value)

		EQ-VAS		EQ-5D	
		Estimate*	p-value	Estimate*	p-value
**Occupation**					
Managers and professionals		−1.518	0.0356	−0.582	0.0209
Clerks		−2.628	0.0030	−0.802	0.0099
Service and sales workers		−0.529	0.4362	−0.663	0.0217
Skilled agricultural, forestry and fishery workers		0.162	0.8720	−1.063	0.1168
Trade workers and elementary occupations		−0.654	0.2373	−0.828	0.0007
Not working		−0.937	0.069	−0.743	0.0251
**Education**					
Elementary school		−0.127	0.8652	−0.747	0.1832
Middle school		−0.471	0.5298	−0.333	0.482
High school graduate		−0.952	0.0407	−0.968	<.0001
College graduate		−1.606	0.0003	−0.532	0.0047
**Family income**					
Q1		−1.302	0.0683	−0.910	0.0587
Q2		−1.106	0.0368	−0.681	0.0155
Q3		−0.732	0.1351	−0.371	0.0934
Q4		−0.884	0.1077	−0.872	0.0003

*All coefficients are adjuted for marital status, sex, smoking status, alcohol consumption, stress, number of chronic diseases, age and year

*All coefficients are the results of hepatitis B patients compared to general population

## Discussion

Because an estimated 240 million people worldwide are chronically infected with HBV, it is important to understand the effects of this disease on patient health and HRQoL [5]. With growing awareness of the implications of chronic liver disease on HRQoL, research has been conducted by several groups into the effects of HBV infection on patient HRQoL. Assessing the health-utility score reported by these patients would allow us to better understand the effects of HBV^+^ status on these patients QoL and would help to improve their care management.

Our study assessed differences of HRQoL in HBV^+^ patients compared to the general population. The proportion of HBV^+^ patients in our study was 16.7 % of total participants, and 33.4 % of the infected patients were not working. We also found that 68.2 % of HBV^+^ patients consumed alcohol, which was similar to the 68.8 % drinking rate in the general population. These results might be considered with respect to patient employment status. In Korea, many employees informally gather to drink alcohol in coworker networks to promote working relationships [23]. This cultural trait might be affected alcohol consumption in both of general population and HBV patients.

In our study, HBV^+^ patients scored lower in HRQoL parameters than did the general population. This observation is likely related to the disease status, wherein HBV patients feel discomfort and fatigue during everyday life activities, which might negatively affect their mental health. These results were similar to those of previous studies on the association between HBV status and patient HRQoL [18, 24–26]. However, we also investigated several additional factors that could potentially affect HRQoL, such as education, family income, and occupation.

In sub-group analysis, according to occupation type, clerks had the lowest scores in EQ-VAS which were even lower than scores of not-working patients. These results contrast with other findings of lowest HRQoL scores in unemployed HBV^+^ patients [27]. This disparity might be due to specific occupational characteristics [28]. Clerks usually deal directly with customers and may feel more emotional stress than other occupations. Because they need to accommodate customer complaints and satisfy supervisor demands, these occupations may have inherently higher stress levels that negatively affect their HRQoL. Also, because working schedules in these occupations are generally inflexible, these patients might not be able to visit the hospital even if they felt sick. Inability to seek timely medical care might further negatively affect these HBV patients’ HRQoL.

Hepatitis-B patients with higher education status had lower HRQoL scores compared to the general population. This might be a result of the social climate in Korea, in which generally it is difficult to people to have sick leave, even though they are cancer [22, 29]. Because, chronic hepatitis B is a disease with no specific visible symptoms, it might be considered a fake illness to other people. If HBV^+^ patients take sick leave, they might encounter social stigmatization because other people may think that these patients were not working faithful and hard. Eventually, this belief might affect HBV^+^ patients’ promotion and they could be excluded from important positions. In this way, the social mood in Korea might preferentially and negatively affect HBV^+^ patients with higher academic status.

We found that high-income HBV^+^ patients had lower HRQoL scores on the EQ-5D survey. Usually, higher-income status would be had more opportunity to management of themselves, through visiting hospital or maintaining good nutrition. However, chronic hepatitis could be lead to liver cancer in the future, it might be affected negatively to patients due to the un-awareness of their disease progression.

This study had limitations. First, it was a cross-sectional design; therefore, causal relationships between HBV^+^ patient HRQoL and socioeconomic status factors could not be definitively established. Second, we did not consider disease progression and severity in HBV+ patients; therefore, the results may not be applicable some patients such as those with resultant cirrhosis or liver cancer. Also, we only considered chronic diseases such as asthma, diabetes, hypertension, dyslipidemia and thyroid lesion because the data used in our study not included other chronic diseases. Finally, we did not assess potential changes in patient occupation type and income after HBV diagnosis, which may have impacted HRQoL. Despite these limitations, our study had several strengths. First, we used data from the Community Health Survey, which ensured use of a reliable nationwide sampling design. Second, we analyzed differences in HRQoL in HBV^+^ patients versus the general population, not only based on HBV status, but also include according to the type of occupation, education history, and family income. The results of our subgroup analyses might help us to better manage HBV^+^ patient care to improve their HRQoL. Third, we used propensity score matching method, it might be reduce bias in estimating effect of quality of life in the HBV patients compared general population [30].

Our findings indicate that HBV^+^ patients have lower HRQoL scores compared to the general population, especially in patients employed as clerks and managers and professionals, and in those with and higher academic and income statuses. These results highlight the important role of health care providers in managing HBV^+^ patient care and educating the general population with accurate information about the disease [31–33]. Having exact information regarding how HBV status affects patient HRQoL is critical to understanding disease impact on everyday activities and attitudes. Although HBV does not manifest visible symptoms of illness, patients still must continuously manage themselves to stop the disease from worsening. Education should include not only pathology details of the disease but also the importance of routine disease screening [33]. It is necessary to provide public education and awareness campaigns that play important roles in promoting HBV screening.

Additionally, companies have to increase employee understanding of the disease and provide regular health inspections. This may help to cultivate the knowledge of the disease and reduce prejudices about HBV^+^ patients. And, superiors in the service industries have to consider giving sick leave to HBV^+^ employees so that they can go to the hospital and check their health condition, when necessary.

## Conclusions

In conclusion, we identified significant relationships between HBV^+^ patient HRQoL and socioeconomic factors such as occupation type, education level, and family income, although further study of these associations is needed. Worldwide efforts to prevent HBV infection have been very successful; now, we have to focus on optimally managing HBV^+^ patient care and improving their HRQoL. To do this, we must acquire accurate information about the disease, including HBV impact on patient lifestyle and sense of well-being. This will facilitate formulating policies and management strategies to mitigate HRQoL declines in HBV^+^ patients.

## Abbreviations

HBV: Hepatitis B virus
HRQoL: Health-related quality of life
